# Characteristics of cytokines/chemokines associated with disease severity and adverse prognosis in COVID-19 patients

**DOI:** 10.3389/fimmu.2024.1464545

**Published:** 2024-11-25

**Authors:** Jianghao Cheng, Haozhen Wang, Chaodan Li, Jianhua Yu, Mingli Zhu

**Affiliations:** ^1^ Department of Open Laboratory Medicine, Hangzhou Xixi Hospital Affiliated to Zhejiang Chinese Medical University, Hangzhou, China; ^2^ Department of General Practice, Jinhua People’s Hospital, Jinhua, China; ^3^ Department of Infectious Diseases, Hangzhou Xixi Hospital Affiliated to Zhejiang Chinese Medical University, Hangzhou, China

**Keywords:** COVID-19, SARS-CoV-2, cytokines, chemokines, prognosis

## Abstract

**Background:**

Cytokines and chemokines as crucial participants in innate immune response play significant roles during SARS-CoV-2 infection, yet excessive immune response exacerbates the severity of COVID-19.

**Purpose:**

This study aims to investigate the involvement of which cytokines/chemokines in the cytokine storm of COVID-19, as well as the changes in cytokine/chemokine levels during the course of COVID-19, simultaneously exploring the diagnostic and prognostic value of the relevant cytokines/chemokines for COVID-19.

**Methods:**

Flow cytometry was employed to detect the levels of cytokines and chemokines in the serum of 50 COVID-19 patients.

**Results:**

Compared with severe COVID-19 patients, the levels of cytokines IL-6, IL-8, IL-10, sCD25, and chemokines IP-10 and MIG in the peripheral blood of non-severe patients were significantly reduced, while only IL-6, IL-10, and IP-10 levels were significantly decreased compared to non-survivors of COVID-19. Meanwhile, serum concentrations of IP-10, MCP-1, sTREM-1, IL-10, and the neutrophil-to-lymphocyte ratio (NLR) in peripheral blood could distinguish between COVID-19 survivors and non-survivors and were significantly associated with mortality. Among them, the concentration of IP-10 was shown to be the most powerful indicator for predicting adverse outcomes in COVID-19 patients (AUC: 0.715); however, its combined detection with the conventional inflammatory marker NLR did not improve the predictive value for adverse outcomes in COVID-19 patients. Additionally, serum IP-10 levels were negatively correlated with peripheral blood NK cell count and total lymphocyte count, while sTREM-1 levels were positively correlated with peripheral blood CD4+ T cell count and CD3+ T cell count. Meanwhile, IL-8 levels were positively correlated with total lymphocyte count in peripheral blood. Finally, the serum levels of cytokines/chemokines in non-survivors of COVID-19 increased significantly before death, while in survivors, they returned to normal levels before discharge.

**Conclusions:**

Severely ill and non-surviving COVID-19 patients exhibit compromised immune function, with significantly higher levels of inflammation, cytokine/chemokine storms, and immune dysregulation compared to non-severe patients. Serum concentrations of IP-10, MCP-1, sTREM-1, and IL-10 levels can serve as biomarkers to predict adverse outcomes in COVID-19.

## 1 Introduction

Previous studies have indicated that cytokines play a crucial role as key participants in the innate immune response during SARS-CoV-2 infection; however, excessive immune responses can exacerbate the severity of COVID-19 (1). This excessive immune-inflammatory response, termed “cytokine storm,” is one of the major causes of acute respiratory distress syndrome (ARDS) and multi-organ failure in COVID-19 patients (2). IL-6, as a biomarker for the prognosis and treatment response of COVID-19 patients, has been extensively studied and applied in clinical practice (3, 4); however, the measurement of individual cytokine levels alone cannot fully reflect the immune status of patients. Therefore, the assessment of multiple cytokine levels is necessary for a comprehensive evaluation, providing valuable insights for the treatment and prognosis of the disease.

Chemokines, as another major component of the inflammatory microenvironment, are a class of small cytokines or signaling proteins secreted by cells. They possess the ability to induce directed chemotaxis of nearby responsive cells, playing a crucial role in attracting leukocytes to sites of infection and contributing significantly to immune-inflammatory responses (5). Previous studies have indicated that chemokines play an important role in modulating the balance between cancerous and non-cancerous cells (6). However, there is still limited data regarding the relationship between the severity of COVID-19 and the dysregulation of chemokines. The overall profile of chemokines in COVID-19 patients of varying severity remains unclear, and there is a lack of relevant biomarkers for predicting patient prognosis.

Therefore, this study aims to investigate which cytokines/chemokines are involved in the cytokine storm of COVID-19. It examines the differences in the levels of 21 cytokines, including IL-2, IL-4, IL-6, IL-10, TNF-α, IFN-γ, IL-17A, IL-1β, IL-5, IL-12p70, IFN-α, IL-8, sCD25, sCD40L, sCD130, sTREM-1, TGF-β, G-CSF, GM-CSF, TGF-A, VEGF, and 5 chemokines, including IP-10, MIG, SDF-1, MCP-1, RANTES, in the serum of non-severe and severe COVID-19 patients. It also explores the changes in cytokines/chemokines during the course of COVID-19 and investigates the diagnostic and prognostic value of relevant cytokines/chemokines for COVID-19.

## 2 Materials and methods

### 2.1 Case source and grouping

This study enrolled 50 COVID-19 patients admitted to Hangzhou Xixi Hospital, Affiliated to Zhejiang Chinese Medical University, from December 2022 to January 2023. The SARS-CoV-2 infection was confirmed via RT-PCR using nasopharyngeal swab samples. And only serum samples were collected for analysis in this study; no tissue samples from deceased patients were obtained. COVID-19 patients were classified into non-severe group (mild or moderate) and severe group (severe or critical) based on clinical diagnosis by physicians and the severity of the patients’ illness. This study was approved by the Ethics Committee of Hangzhou Xixi Hospital, Affiliated to Zhejiang Chinese Medical University. The clinical classification and diagnostic criteria of COVID-19 refer to the “Diagnosis and Treatment Protocol for Novel Coronavirus Pneumonia (Trial Version 9)” issued by the National Health Commission of the People’s Republic of China.

### 2.2 Serum cytokine quantification

#### 2.2.1 Cytokine measurement platform

The cytokine and chemokine levels in serum were quantified using flow cytometry (BD FACS Canto, BD Biosciences). The specific panel of cytokines measured includes IL-2、IL-4、IL-6、IL-10、TNF-α、IFN-γ、IL-17A、IL-1β、IL-5、IL-12p70、IFN-α、IL-8、sCD25、sCD40L、sCD130、sTREM-1、TGF-β、G-CSF、GM-CSF、TGF-A、VEGF、IP-10、MIG、SDF-1、MCP-1、RANTES.

#### 2.2.2 Reagents and kits

The assay kits used for cytokine detection were procured from CEGER (Jiangxi, China), specifically the Cytokine and Chemokine combined detection kit, which includes a comprehensive set of reagents for multiplexed flow cytometry detection.

### 2.3 Procedure

#### 2.3.1 Sample preparation

Serum samples were thawed at room temperature and centrifuged at 1,500 rpm for 5 minutes to remove debris.

#### 2.3.2 Cytokine detection

For each serum sample, capture the microsphere mixture of 25μL from the kit and add it into the test tube, swirl and shake for 30 seconds; Then put the test tube into a centrifuge, 300g/min, centrifuge at room temperature for 5 minutes; After centrifugation, the supernatant was carefully absorbed and discarded with a pipette, then 25μL microsphere buffer was taken to re-suspend the microsphere, and after swirling for 5 seconds, the microsphere was incubated for 30 minutes away from light. Swirl and mix the incubated trapped microsphere mixture for 30 seconds and add 25μL into each experimental tube. The melted 25μL serum sample to be tested was added to the test tube in step 2 and thoroughly mixed in the swirl. Add 25μL of fluorescence detection reagent into each tube and gently shake. The tube was wrapped in tin foil and incubated for 2.5 hours in the dark at room temperature. After incubation, 1mL of PBS washing solution was added into the test tube, thoroughly mixed, centrifuged at 300g/min in room temperature for 5 minutes, and the supernatant was discarded. Add 500μL PBS solution again and prepare for the machine after shock suspension. Data were read by BD FACSCanto flow cytometry.

#### 2.3.3 Chemokine detection

Add 25μL of each serum sample from the diluent of each kit, the mixed solution of captured microspheres and the antibody detection reagent into the test tube, and swirl and mix well. The melted 25μL serum sample was added to the test tube in step 1 and mixed by shock. Wrap the pipe with tin foil and shock at 2000rpm at room temperature for 2 hours away from light. Add 25μL of SA-PE into the test tube. Wrap the pipe with tin foil and shake at 2000rpm at room temperature for 0.5 hours away from light. Centrifuge 250g/min for 5 minutes, absorb the supernatant with a pipette and discard it. Add 250μL PBS buffer into the test tube, swirl and mix for 30 seconds. Centrifuge 250g/min for 5 minutes, absorb the supernatant with a pipette and discard it. Add 500μL PBS buffer into the test tube, swirl and mix for 30 seconds, ready for the machine. Data were read by BD FACSCanto flow cytometry.

### 2.4 Statistical analysis

Statistical analysis was performed using SPSS 23.0 software. For continuous variables, normality was assessed, and normally distributed data were presented as mean ± standard deviation, analyzed using paired *t*-tests. Non-normally distributed data were expressed as median (interquartile range) [M (P25, P75)] and analyzed using non-parametric Mann-Whitney U tests. Categorical variables were presented as frequencies and percentages, analyzed using chi-square tests or Fisher’s exact tests. Correlation analysis between variables was conducted using Spearman’s correlation test. A significance level of *P* < 0.05 was considered statistically significant for all analyses. The diagnostic value of selected parameters for distinguishing between deceased and surviving COVID-19 patients was evaluated using Receiver Operating Characteristic (ROC) analysis and the Area Under the Curve (AUC). Kaplan-Meier analysis assessed the survival rates of COVID-19 patients, with differences between survival curves compared using the Log Rank test.

## 3 Results

### 3.1 Demographical characteristics

This study included a total of 50 COVID-19 patients admitted to the Hangzhou Xixi Hospital, Affiliated to Zhejiang Chinese Medical University from December 2022 to January 2023. Among them, there were 19 cases (38.0%) in the non-severe group, with 10 males and 9 females, and 31 cases (62.0%) in the severe group, with 19 males and 12 females. The non-survivor group comprised a total of 17 cases, with 13 males and 4 females. The median age of all patients was 81 years, with the youngest being 27 years old and the oldest 98 years old. Among the non-severe group, 16 patients (84.2%) were aged 60 or older, while among the severe group, 29 patients (93.5%) were in this age category, and among the non-survivor group, 15 patients (88.2%) were aged 60 or older. Common underlying conditions included diabetes (n=12, 24.0%), coronary heart disease (n=11, 22.0%), hypertension (n=22, 44.0%), and there were 11 patients (22.0%) with a history of smoking. Only one case (2.0%) had a history of exposure in an epidemic area, while 49 cases (98.0%) did not. Specific clinical baseline characteristics of the patients are shown in **Table 1**.

**Table 1:** Demographical characteristics of COVID-19 patients.

Baseline characteristic	Non-severe group (n=19)	severe group (n=31)	Total (n=50)	*p-value*
Sex (M/F)	10/9	19/12	29/21	0.570
Year	71.1 ± 15.7	81.6 ± 9.1	77.6 ± 13.0	0.004
Weight (Kg)	61.8 (57.6-65.8)	65.0 (46.5-70.0)	62.0 (55.0-69.5)	0.466
Height (m)	1.61 (1.57-1.70)	1.65 (1.50-1.72)	1.62 (1.56-1.71)	0.817
Smoking (n, %)	6 (31.6)	5 (16.1)	11 (22.0)	0.293
Underlying disease (n, %)	7 (36.8)	22 (71.0)	29(58.0)	0.022
Diabetes (n, %)	0 (0)	12 (38.7)	12 (24.0)	0.002
Coronary disease (n, %)	2 (10.5)	9 (29.0)	11 (22.0)	0.170
Hypertension (n, %)	7 (5.3)	15 (48.4)	22 (44.0)	0.559
Contact history (n, %)	1 (5.3)	0 (0.0)	1 (2.0)	0.380
Non-contact history (n, %)	18 (94.7)	31 (100.0)	49 (98.0)	
Ending event (n, %)				0.007
Survive	17 (89.5)	16 (51.6)	33 (66.0)	
Death	2 (10.5)	15 (48.4)	17 (34.0)	

Continuous data are expressed as median and interquartile range M (P25~P75) or mean ± standard deviation (x ± s). Categorical data are presented as n (%). P-value < 0.05 indicates statistical significance.

### 3.2 Differential expression profile of cytokines/chemokines in COVID-19 patients of varied severity

**Figure 1:**
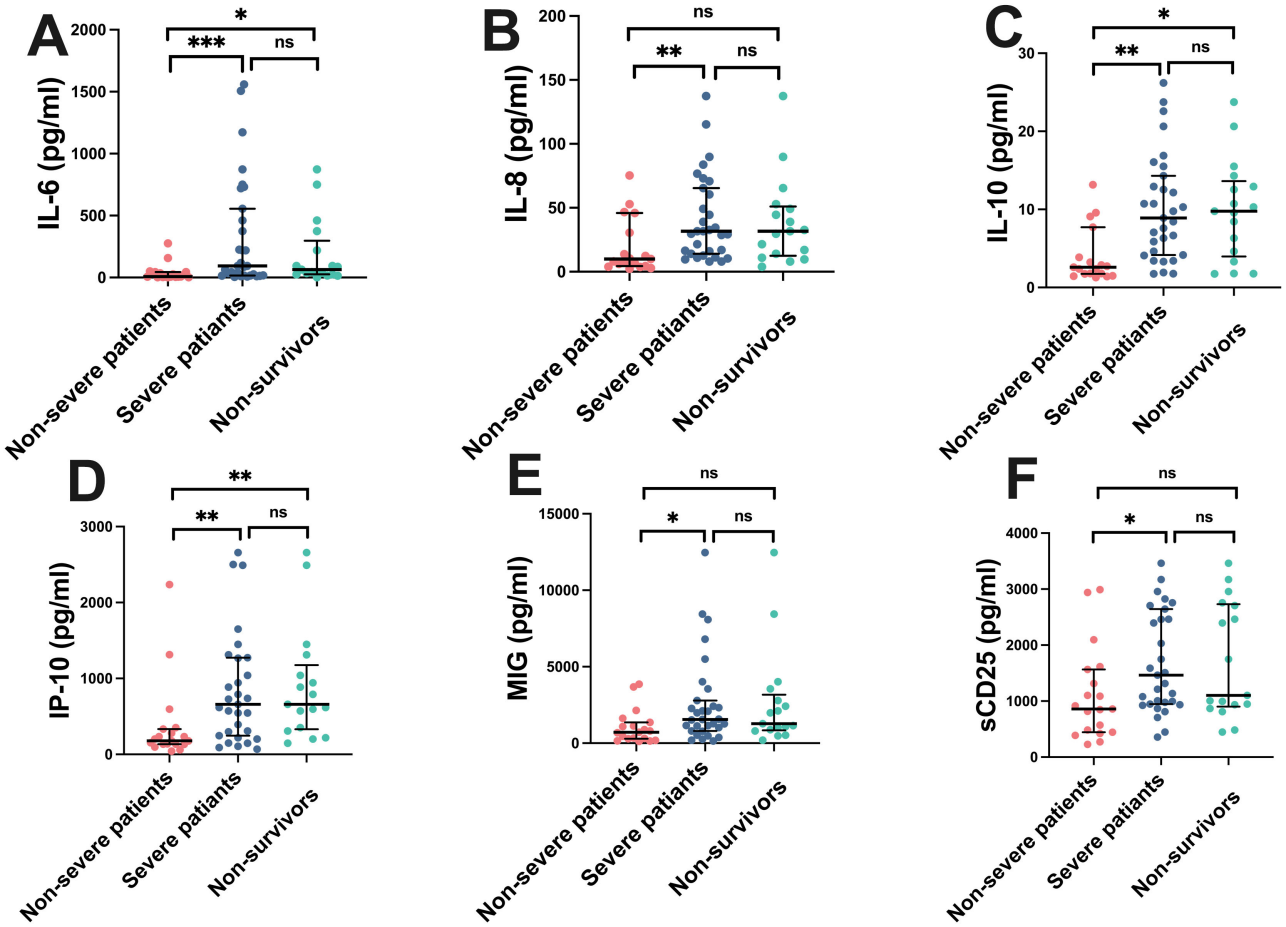
Cytokine/Chemokine levels in COVID-19 patients of different severity. Fifty COVID-19 patients were divided into three groups: non-severe group, severe group, and non-survivor group. Serum concentrations of IL-6, IL-8, IL-10, IP-10, MIG, and sCD25 were analyzed. Median values with ranges were provided. *P < 0.05, **P < 0.01, ***P < 0.001, ns indicates no statistical difference. **(A)** IL-6, **(B)** IL-8, **(C)** IL-10, **(D)** IP-10, **(E)** MIG, **(F)** sCD25.

To investigate the differences in cytokine/chemokine expression levels among COVID-19 patients of varying severity, we further analyzed the expression profiles of cytokines/chemokines in COVID-19 patients based on disease severity (**Figure 1**). Statistical analysis revealed that levels of cytokines IL-6, IL-8, IL-10, sCD25, and chemokines IP-10, and MIG in the peripheral blood of severe COVID-19 patients were significantly higher than those in non-severe patients (P < 0.05). However, the differences in levels of other cytokines such as IL-2, IL-4, TNF-α, IFN-γ, IL-17A, IL-1β, IL-5, IL-12p70, IFN-α, sCD40L, sCD130, sTREM-1, TGF-β, G-CSF, GM-CSF, TGF-A, VEGF, and chemokines SDF-1, MCP-1, RANTES showed no statistical significance (P > 0.05). In the comparison between non-survivors and non-severe patients, levels of IL-6, IL-10, and IP-10 in the peripheral blood of non-survivors were significantly higher than those in non-severe patients (P < 0.05), while the differences in IL-8, MIG, and sCD25 levels showed no statistical significance (P > 0.05). There were no statistically significant differences in IL-6, IL-8, IL-10, sCD25, IP-10, and MIG levels between severe patients and non-survivors (P > 0.05). These results suggest that serum levels of IL-6, IL-8, IL-10, IP-10, MIG, and sCD25 upon admission of COVID-19 patients may be associated with disease severity. However, differences in the serum levels of other cytokines/chemokines among patients with different severity levels do not have statistical significance.

### 3.3 Predictive efficacy of IP-10, MCP-1, sTREM-1, and IL-10 concentrations for COVID-19 prognosis

**Figure 2:**
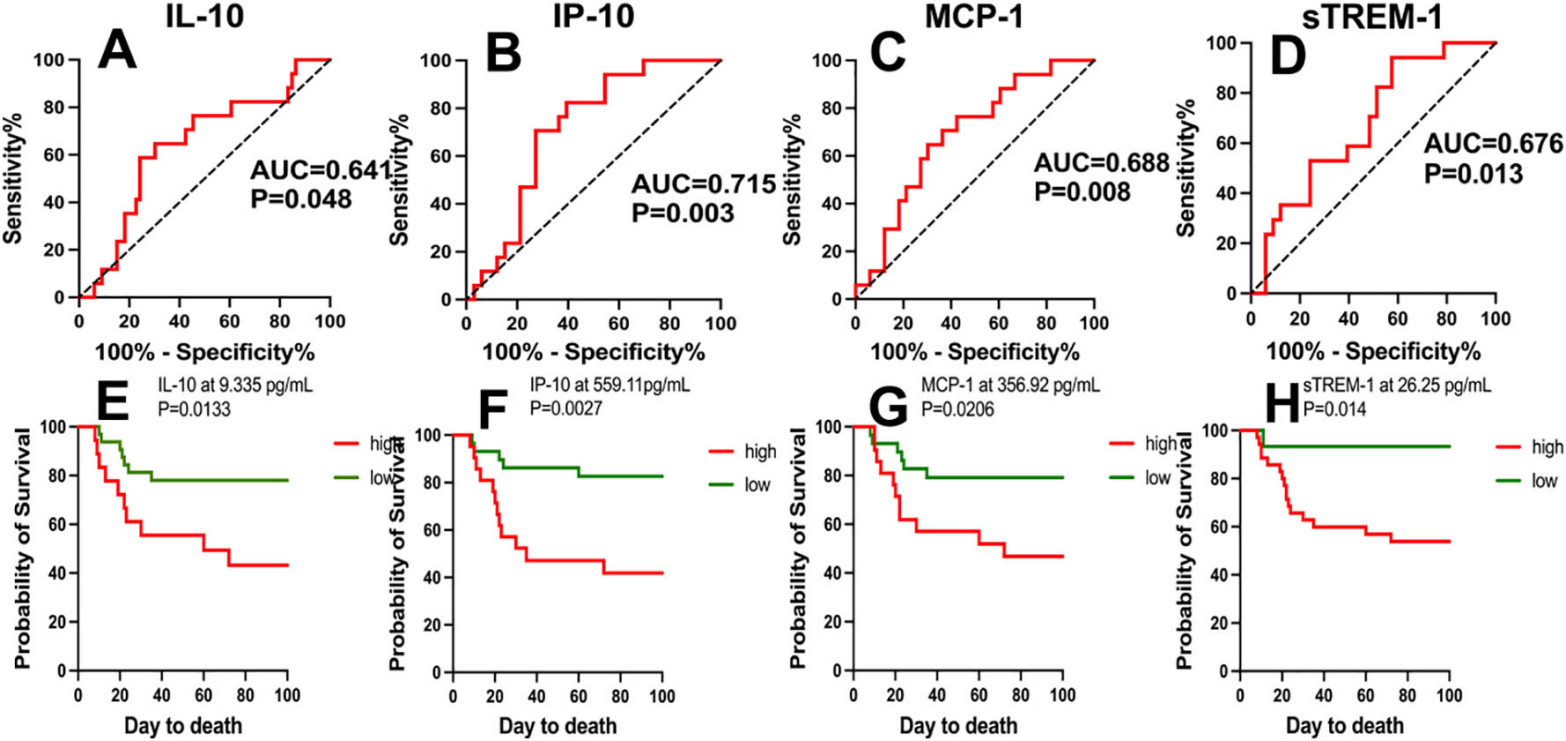
ROC curves of IP-10, MCP-1, sTREM-1, and IL-10 concentrations and Kaplan-Meier survival analysis. **(A-D)** The performance of ROC curves of IP-10, MCP-1, sTREM-1, and IL-10 in predicting adverse clinical outcomes in COVID-19 patients. **(E-H)** Kaplan-Meier survival analysis tested the survival function using critical values obtained from the ROC curves of IP-10, MCP-1, sTREM-1, and IL-10. P values < 0.05 were considered statistically significant.

We analyzed the predictive value of various cytokines/chemokines for the prognosis of COVID-19 patients. Positive samples were COVID-19 non-survivors, and negative samples were COVID-19 survivors. ROC analysis revealed that IP-10, MCP-1, sTREM-1, and IL-10 had significant value in distinguishing COVID-19 patients with different clinical outcomes. The sensitivity, specificity, and AUC of IP-10 at the critical value (559.11 pg/mL) were 70.6%, 72.2%, and 0.715 (95% CI: 0.586-0.843), respectively, with P=0.003; for MCP-1, they were 70.6%, 69.7%, and 0.688 (95% CI: 0.560-0.816) at the critical value (356.92 pg/mL), respectively, with P=0.008; for sTREM-1, they were 94.1%, 42.4%, and 0.676 (95% CI: 0.546-0.805) at the critical value (26.25 pg/mL), respectively, with P=0.013; and for IL-10, they were 58.8%, 75.6%, and 0.641 (95% CI: 0.504-0.777) at the critical value (9.335 pg/mL), respectively, with P=0.048. Subsequently, we used Kaplan-Meier analysis to test the survival function obtained from the critical value points on the ROC curve. Serum concentrations of IP-10, MCP-1, sTREM-1, and IL-10 above 9.335 pg/mL, 559.11 pg/mL, 356.92 pg/mL, and 26.25 pg/mL, respectively, were significantly associated with mortality (P<0.05). Patients with cytokine/chemokine levels above the critical values mostly had poor prognosis, with a significantly increased mortality rate within 40 days, as shown in **Figure 2**. From the above analysis, it can be concluded that the ROC curves of IP-10, MCP-1, sTREM-1, and IL-10 can distinguish between COVID-19 survivors and non-survivors and may have predictive value for the prognosis of COVID-19 patients. Among them, IP-10 had the largest AUC, indicating that IP-10 had the best predictive efficacy for the prognosis of COVID-19 patients, with a sensitivity and specificity of 70.6% and 72.2%, respectively.

### 3.4 Comparison of predictive efficiency of cytokines/chemokines and common inflammatory markers for prognosis in COVID-19 patients

**Figure 3:**
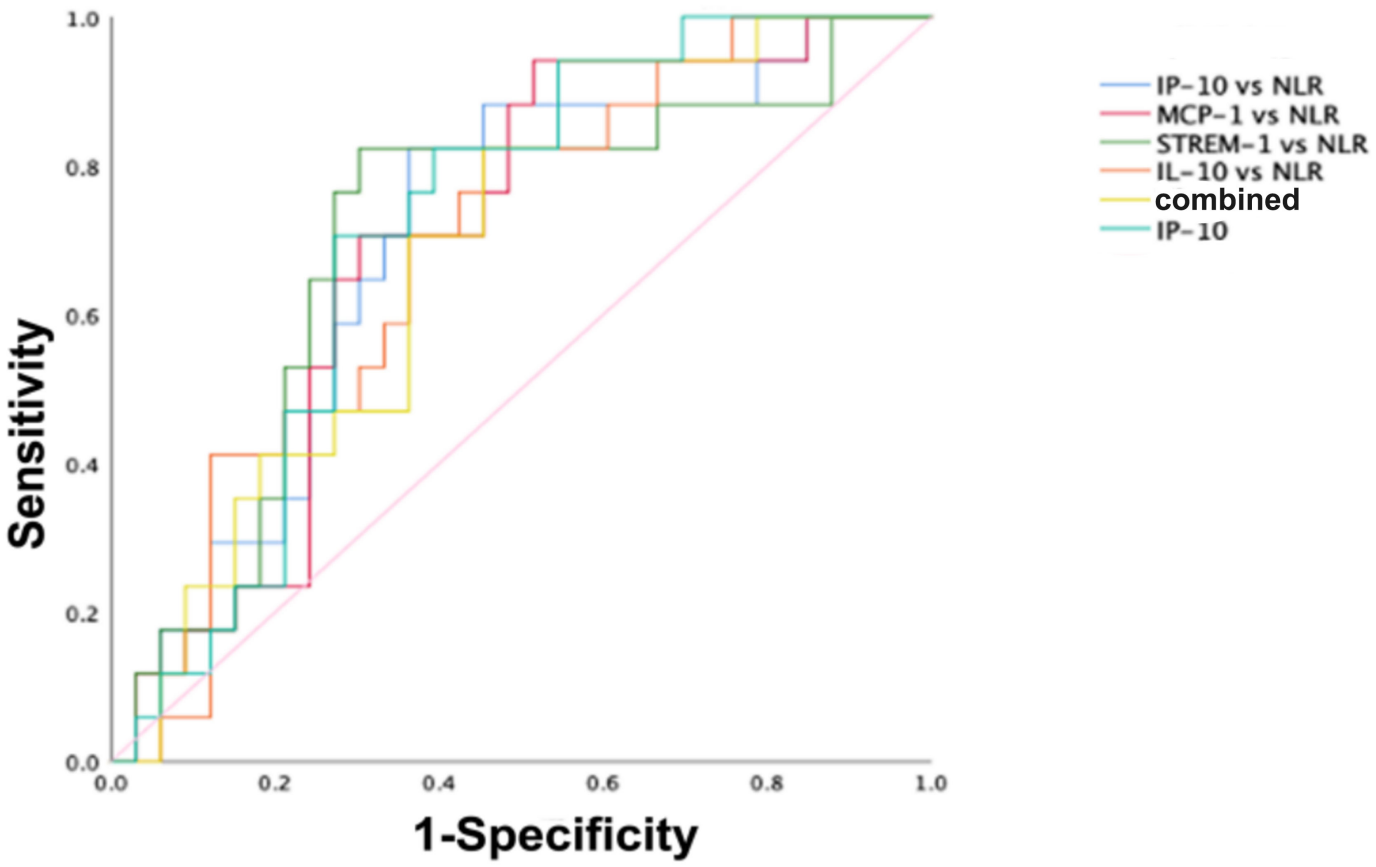
ROC curves for distinguishing survivors from non-survivors in COVID-19 patients. The ROC curves for IP-10, IP-10 combined with NLR, MCP-1 combined with NLR, sTREM-1 combined with NLR, IL-10 combined with NLR, and the combined detection of the five indicators in predicting the mortality outcome of COVID-19 patients.

To further evaluate whether the diagnostic value of cytokines/chemokines in surviving and non-surviving COVID-19 patients is superior to that of other common inflammatory markers, and whether the combined diagnostic value is improved compared to single markers, we included neutrophil-to-lymphocyte ratio (NLR), lymphocyte-to-monocyte ratio (LMR), platelet-to-lymphocyte ratio (PLR), and inflammatory markers such as SAA and CRP in the comparative study. The ROC analysis results are shown in **Table 2**. It was found that, except for NLR, LMR, PLR, SAA, and CRP showed no diagnostic value in distinguishing between surviving and non-surviving COVID-19 patients (P > 0.05). The AUC of NLR was 0.693, lower than that of IP-10 but higher than the AUCs of MCP-1, sTREM-1, and IL-10. The combined diagnostic model was established through binary logistic regression analysis, and the results showed that the combined diagnosis did not improve the predictive value of COVID-19 mortality outcome, as shown in **Figure 3**. IP-10 had the largest AUC among all single cytokines/chemokines and common inflammatory markers, and a serum IP-10 concentration of 559.11 pg/mL was the optimal cutoff value for predicting death in COVID-19 patients (sensitivity = 70.6%, specificity = 72.2%). The AUC of sTREM-1 combined with NLR was second only to that of IP-10 alone, at 0.704. However, the combined detection of IP-10, MCP-1, sTREM-1, IL-10, and NLR had the smallest AUC, at only 0.688.

**Table 2:** ROC analysis results of common inflammatory markers for distinguishing between surviving and non-surviving COVID-19 patients.

Common inflammatory index parameters	AUC	95% CI	*p-value*
NLR	0.693	0.530-0.857	0.026
LMR	0.627	0.207-0.538	0.143
PLR	0.622	0.456-0.788	0.161
SAA (mg/L)	0.574	0.411-0.736	0.401
CRP (mg/L)	0.620	0.458-0.783	0.167

P-value < 0.05 indicates statistical significance.

Based on the above analysis, the diagnostic value of the chemokine IP-10 in surviving and non-surviving COVID-19 patients is superior to that of other common inflammatory markers. The combination detection of MCP-1, sTREM-1, and IL-10 with NLR showed an improved AUC compared to the individual detection of MCP-1, sTREM-1, and IL-10, with values of 0.697, 0.704, and 0.690, respectively. However, the combined detection of IP-10, MCP-1, sTREM-1, IL-10, and NLR did not improve the predictive value for COVID-19 mortality outcome.

### 3.5 Dynamic changes of serum cytokines/chemokines in COVID-19 patients upon admission and discharge

**Figure 4:**
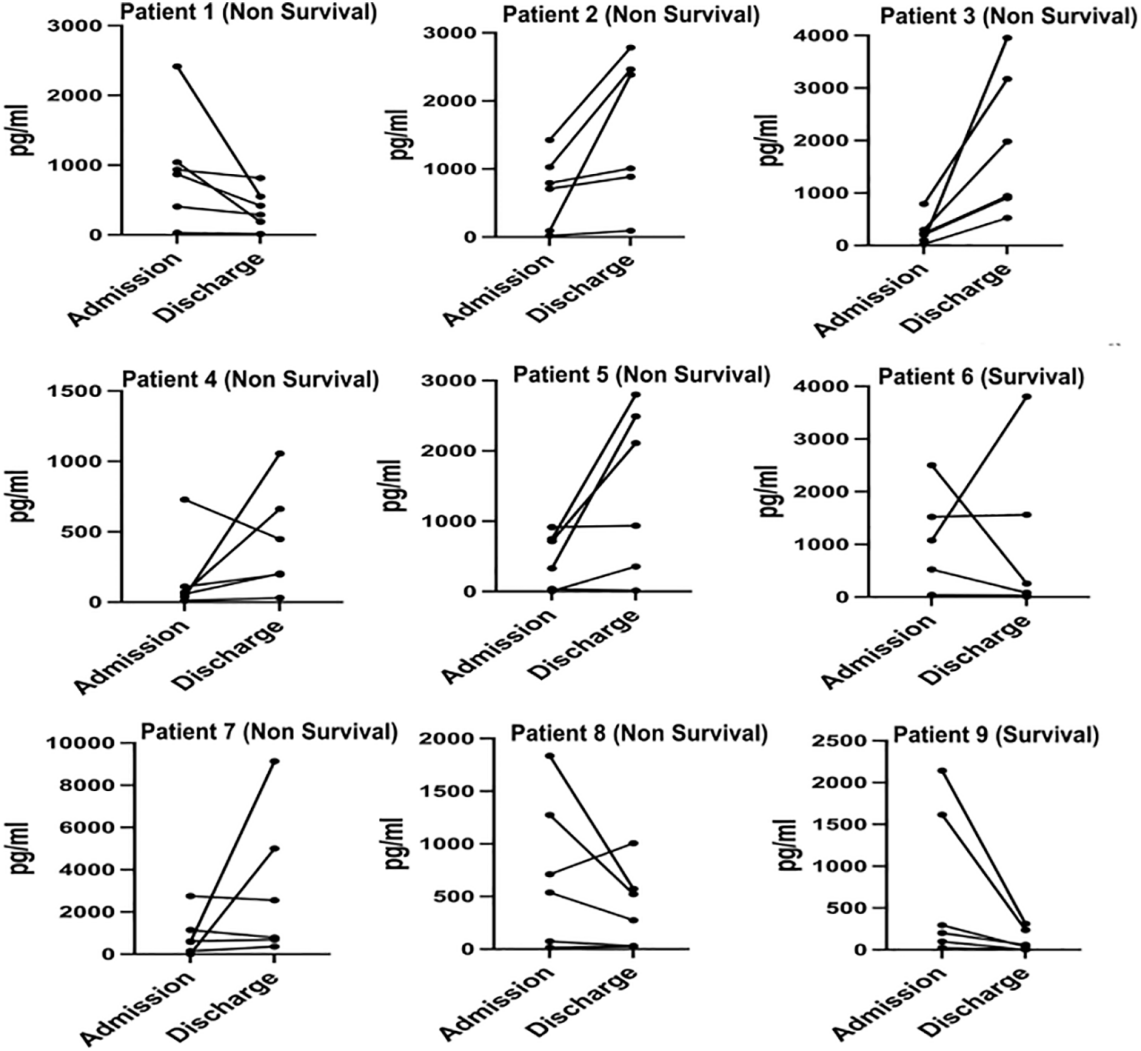
Dynamic profile of cytokine/chemokine levels in COVID-19 survivors and non-survivors.

**Figure 5:**
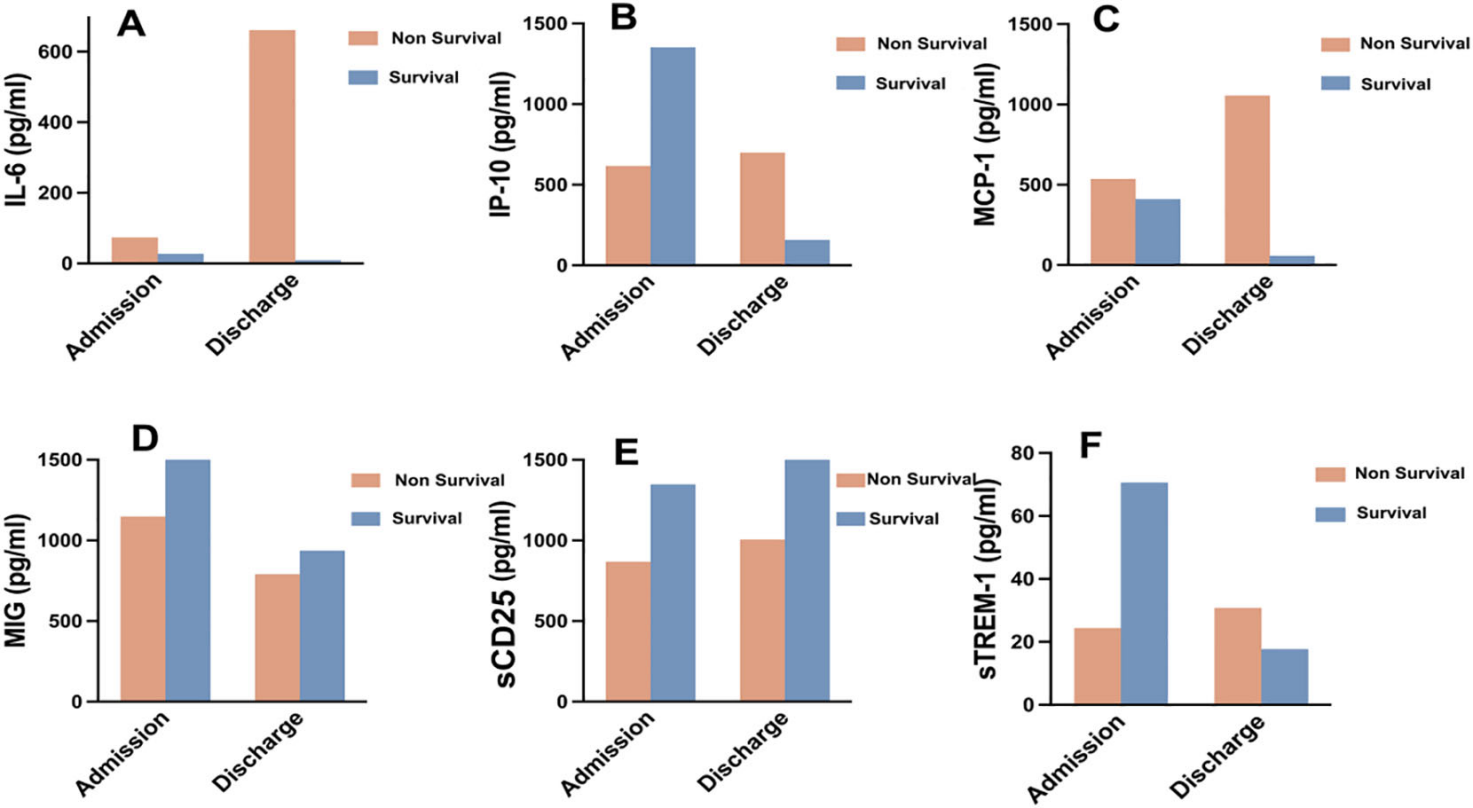
Expression characteristics of selected cytokines/chemokines in COVID-19 survivors and non-survivors at admission and before discharge. **(A)** IL-6, **(B)** IP-10, **(C)** MCP-1, **(D)** MIG, **(E)** sCD25, **(F)** sTREM-1.

We monitored and detected the expression levels of IP-10, MCP-1, MIG, sCD25, sTREM-1, and IL-6 in the serum of 9 COVID-19 patients upon admission and before discharge. Among them, 2 cases were survivors, and 7 cases were non-survivors. The dynamic changes of these cytokines/chemokines in each patient’s serum are shown in **Figure 4**. The results indicate that compared to admission, the levels of most cytokines/chemokines in the serum of 5 out of 7 non-survivors tended to increase before death, while in the remaining 2 non-survivors, most cytokines/chemokines showed a decreasing trend. In contrast, the levels of most cytokines/chemokines in the serum of the 2 survivors decreased before discharge. The changes in the expression levels of each cytokine/chemokine between admission and before discharge are illustrated in **Figure 5**. The results show that in non-survivors, the serum levels of IL-6, IP-10, MCP-1, sCD25, and sTREM-1 increased before death, with the serum concentrations before admission and before discharge being IL-6 [74.0 (11.3-203.5) pg/mL vs 903.8 (355.2-5000) pg/mL], IP-10 [616.3 (230.6-1041.1) pg/mL vs 699.0 (203.2-942.2) pg/mL], MCP-1 [536.7 (99.6-748.4) pg/mL vs 1055.4 (288.0-3954.3) pg/mL], sCD25 [868.0 (728.3-1029.4) pg/mL vs 1005.8 (448.1-2552.2) pg/mL], and sTREM-1 [(39.8 ± 17.6) pg/mL vs (152.8 ± 78.3) pg/mL], while the serum concentration of MIG decreased before death, being [1147.5 (297.1-1426.8) pg/mL vs 790.4 (522.7-2111.2) pg/mL]. In survivors, the serum levels of IL-6, IP-10, MCP-1, MIG, and sTREM-1 decreased before recovery, with the serum concentrations before admission and before discharge being IL-6 (27.0 pg/mL vs 9.03 pg/mL), IP-10 (1351.3 pg/mL vs 157.8 pg/mL), MCP-1 (821.4 pg/mL vs 58.715 pg/mL), MIG (1833.5 pg/mL vs 936.2 pg/mL), and sTREM-1 (70.6 pg/mL vs 17.7 pg/mL), while the serum concentration of sCD25 increased during the recovery period, being (1348.1 pg/mL vs 2019.8 pg/mL).

These results suggest that in non-survivors of COVID-19, the levels of cytokines/chemokines in serum increased significantly before death, with IL-6 showing the most significant increase. In this cohort, a critically ill patient deteriorated and died 18 days after admission, with a serum IL-6 concentration as high as 2384 pg/mL on the day before death. Although our study has a limited number of cases, it underscores the importance of IL-6 as a predictor of COVID-19 disease severity. In contrast, in survivors of COVID-19, the levels of cytokines/chemokines in serum decreased significantly before recovery, and the serum IL-6 concentration returned to normal levels before discharge.

### 3.6 Correlation between expression levels of several cytokines/chemokines and subsets of lymphocytes

**Figure 6:**
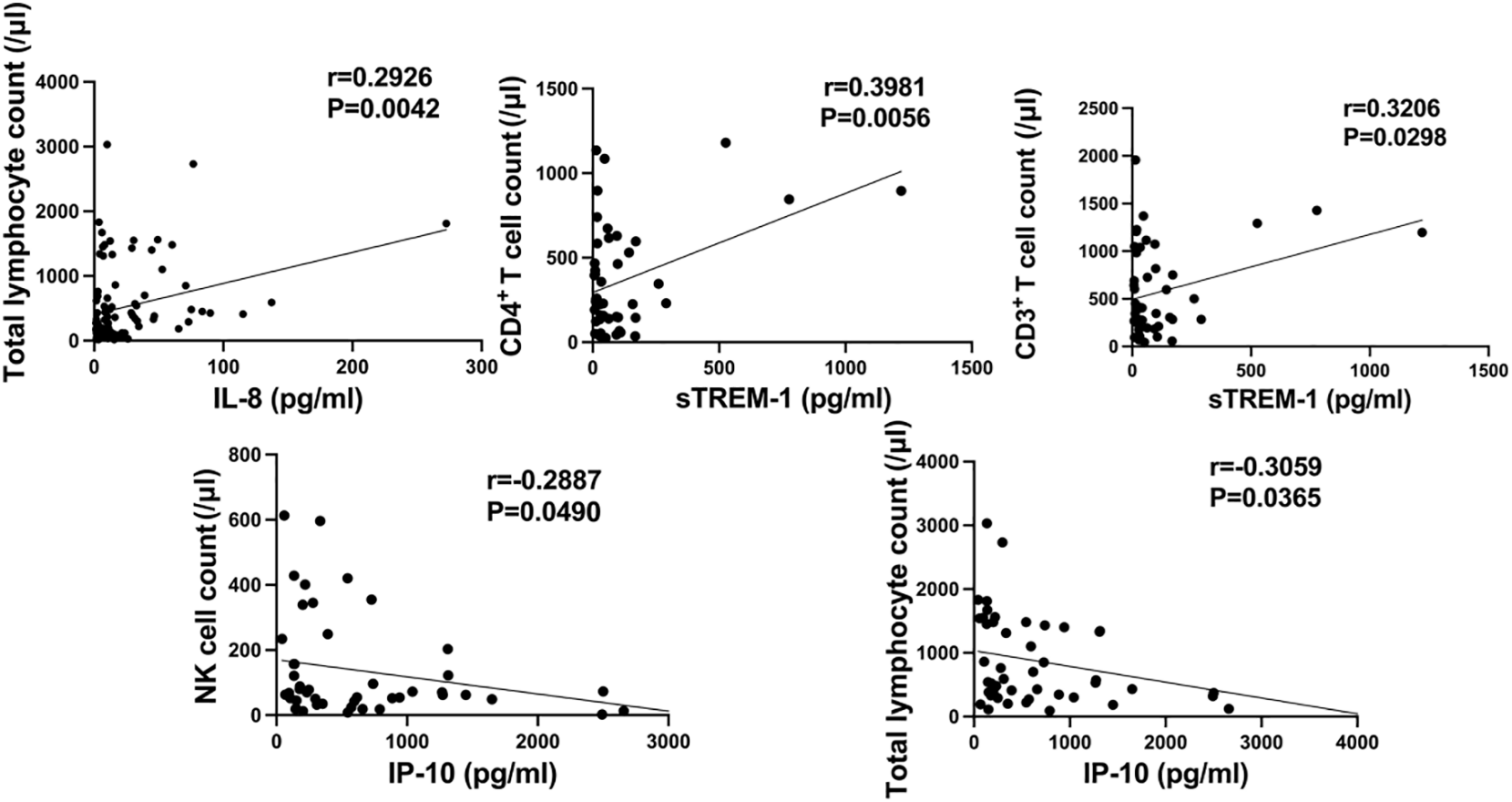
Correlation between serum levels of IP-10, sTREM-1, IL-8, and lymphocyte counts.

We investigated the correlation between the levels of IL-6, IL-8, IL-10, IP-10, MCP-1, MIG, sCD25, sTREM-1, and the counts of T cells, B cells, and NK cells, respectively. As shown in **Figure 6**, the serum levels of IP-10 were negatively correlated with the peripheral blood counts of NK cells and total lymphocytes (r=-0.2887, P=0.0490; r=-0.3059, P=0.0365); the serum levels of sTREM-1 were positively correlated with the counts of CD4+ T cells and CD3+ T cells in peripheral blood (r=0.3981, P=0.0056; r=0.3206, P=0.0298); the serum levels of IL-8 were positively correlated with the total lymphocyte count in peripheral blood (r=0.2926, P=0.0042). No correlation was found between the levels of other cytokines/chemokines and lymphocyte counts.

## 4 Discussion

Cytokines, as a class of low-molecular-weight soluble proteins, play a crucial role in maintaining normal immune function and are involved in various pathological processes such as inflammation, tissue repair, coagulation, and fibrosis. However, excessive production of cytokines due to immune dysfunction can cause harm to the body, leading to a condition known as cytokine storm. The phenomenon of cytokine storm has been observed in autoimmune inflammatory diseases, sepsis, primary and secondary hemophagocytic lymphohistiocytosis, and other pathological conditions (7), and has recently been identified as a key factor in the progression to severe COVID-19 (24). With the rapid increase in the number of severe COVID-19 cases, intensive care unit (ICU) treatment has become a scarce resource. Early identification of severe forms of COVID-19 and prognostic indicators are crucial for saving critically ill patients and conserving medical resources. Therefore, this study investigates the expression of cytokines/chemokines in COVID-19 patients, focusing on their role in assessing the severity and prognosis of the disease, providing valuable insights for clinical diagnosis and treatment.

Growing research data suggests that the severity of COVID-19 is linked with higher levels of inflammatory mediators, such as cytokines, chemokines, tumor necrosis factor, C-reactive protein, ferritin, and D-dimers (8). This study found that the levels of cytokines IL-6, IL-8, IL-10, sCD25, and chemokines IP-10, and MIG in the peripheral blood of severe COVID-19 patients were significantly higher than those in non-severe patients (P < 0.05). This is consistent with reports by Liu Y (9) and Huang C (10). However, no differences were observed in the levels of cytokines IL-2, IL-4, TNF-α, IFN-γ, IL-17A, IL-1β, IL-5, IL-12p70, IFN-α, sCD40L, sCD130, sTREM-1, TGF-β, G-CSF, GM-CSF, TGF-A, VEGF, and chemokines SDF-1, MCP-1, and RANTES between severe and non-severe COVID-19 patients. Severe patients with underlying diseases were significantly more prevalent than non-severe patients, and the mortality rate was significantly higher (P < 0.05). These data suggest that severe COVID-19 patients have more severe disease conditions, and the severity of the disease may be related to the release of cytokines/chemokines, indicating the potential occurrence of cytokine storms in patients infected with SARS-CoV-2.

This study reveals that IP-10, induced by IFN-γ, serves as a crucial chemokine facilitating the transportation of immune cells to sites of inflammation, playing a pivotal role in the host’s defense against viral infections (11) Previous research suggests that IP-10 may serve as a key inflammatory factor predicting the severity of diseases caused by H7N9 avian influenza virus, respiratory syncytial virus, human immunodeficiency virus (HIV), and hepatitis B virus infections (12–15). In our study, we found that serum levels of IP-10 are associated with the severity of diseases caused by SARS-CoV-2 infection, with higher levels observed in severe patients compared to non-severe ones, and IP-10 can help distinguish between survivors and non-survivors. This finding is consistent with previous research highlighting the critical role of cytokines in disease progression (16, 17).Therefore, IP-10 may serve as a prognostic biomarker of certain clinical value in COVID-19. Additionally, we observed a significant negative correlation between serum IP-10 levels and peripheral blood NK cell count and total T cell count, suggesting that exposure to persistent high levels of IP-10 may lead to a decrease in NK cell and T cell numbers. The decreased function and quantity of T cells associated with IP-10 might be a result of reduced calcium signaling pathway and p38 mitogen-activated protein kinase expression (18), and blockade of IP-10 has been shown to increase T cell numbers (19). Similarly to IP-10, MIG, as another IFN-γ-induced chemokine, is a hallmark of host involvement in Th1-type immune responses, playing a crucial role in the initiation and maintenance of various diseases (20). Research suggests that MIG may play a critical role in regulating major pathological events during chronic hepatitis C (21). MCP-1, being an overall chemotactic factor, recruits macrophages for immune responses, which along with its receptors (such as CCR2, ACKR1, and ACKR2), significantly impact diseases across different systems like cancer and diabetes (22). sCD25 is typically regarded as a biomarker for diseases characterized by T cell expansion, and elevated sCD25 levels are associated with enhanced antigen specific Th17 responses (23). TREM-1, first discovered on circulating neutrophils and monocytes, upon activation in neutrophils and monocytes, triggers the release of pro-inflammatory cytokines and chemokines (24). Elevated levels of sTREM-1 have been associated with increased mortality rates in patients with septic shock (25). In our study population, we found that serum levels of MIG and sCD25 were significantly higher in severe patients compared to non-severe ones, and MCP-1 and sTREM-1 aided in distinguishing between COVID-19 survivors and non-survivors, which is similar to the findings reported by Zhang et al. (26). However, some studies have found decreased levels of chemokines such as MIG and IP-10 in the serum of severe COVID-19 patients, which may be limited by the small sample size of their studies (27). Furthermore, we observed a positive correlation between serum sTREM-1 levels and peripheral blood CD3^+^ T cell and CD4^+^ T cell counts. This might be attributed to exacerbated pulmonary infections leading to elevated serum sTREM-1 levels, which subsequently activate lymphocytes (28). The positive correlation between T cell counts and serum sTREM-1 concentration seems to explain the protective effect of increased T cells in COVID-19 patients. T cell dysregulation is a characteristic feature of COVID-19 (29), and in our study, we found that T cells were significantly lower in severe COVID-19 patients compared to non-severe ones, consistent with the findings reported by Song JW et al (30). As one of the immunosuppressive agents, the elevated levels of sCD25 associated with excessive T cell activation in severe COVID-19 patients’ serum may account for this phenomenon. However, we did not observe a relationship between sCD25 and T cell counts (r=0.2659, P=0.0741), which may be due to our current small sample size.

The pathogenesis of COVID-19 involves intense inflammatory responses and encompasses a complex array of mediators, including IL-6, IL-8, and IL-10. These pleiotropic cytokines are produced at sites of tissue inflammation and released into circulation by various immune cells during acute organ injury (31). Among these, IL-6 serves as a primary pro-inflammatory mediator that induces acute-phase responses, leading to local and systemic changes in the body, including fever, leukocyte recruitment, and activation (32). IL-8, produced by macrophages and stromal cells exposed to inflammatory stimuli, is considered a major factor in the local accumulation of neutrophils (33). IL-10, possessing anti-inflammatory properties, plays a pivotal role in infection by limiting immune responses to pathogens, thereby preventing damage to the host (34). In our study, we observed significantly elevated levels of IL-6, IL-8, and IL-10 in COVID-19 severe patients compared to non-severe patients, and IL-10 was able to distinguish between survivors and non-survivors of COVID-19, consistent with reports by Diao and Han et al. (35, 36). This suggests that the levels of these cytokines are associated with the severity and prognosis of the disease, reflecting a more pronounced immune dysregulation in severe COVID-19 patients compared to non-severe patients, leading to increased release of inflammatory mediators. Additionally, we found a positive correlation between serum IL-8 levels and peripheral blood total lymphocyte count, indicating that elevated IL-8 levels may lead to an increase in total lymphocyte count, potentially enhancing the body’s ability to combat viral infections.

In this study, we observed changes in the levels of serum cytokines/chemokines in COVID-19 patients from symptom onset upon admission to recovery or death. Among COVID-19 non-survivors, 71.4% exhibited a substantial increase in serum cytokine/chemokine levels prior to death, whereas survivors showed a marked decrease in cytokine/chemokine levels before recovery. The dynamic changes in cytokines/chemokines among non-survivors were as follows: IL-6 (Δ =829.8), IP-10 (Δ =82.7), MCP-1 (Δ =518.7), sCD25 (Δ =137.8), sTREM-1 (Δ =113.0), MIG (Δ =-357.1). These cytokines/chemokines are involved in innate immune responses, with IL-6 exhibiting the most prominent increase. While IL-6 is considered an immunosuppressive cytokine that helps prevent excessive inflammation, high levels of IL-6 in the serum of non-surviving COVID-19 patients may signal an overactive immune response, potentially leading to adverse outcomes. Our study suggests that monitoring changes in cytokine/chemokine levels before and after treatment can indicate disease progression if levels relatively increase or improvement if levels decrease. Moreover, during pathogenic SARS-CoV-2 infection, the severe cytokine storm causing pathological immune damage may be the true “killer” of critically ill patients.

Currently, there is still no consensus on the critical values of cytokine-based biomarkers in assessing disease prognosis. In this study, we found that IP-10, MCP-1, sTREM-1, and IL-10 have significant value in distinguishing COVID-19 patients with different clinical outcomes. Among them, IP-10 had the largest AUC of 0.715, indicating that IP-10 is the most effective in evaluating the clinical prognosis of COVID-19 patients, with its predictive efficacy superior to that of the common inflammatory marker NLR. Furthermore, the combined diagnosis of these cytokines/chemokines and NLR did not improve the predictive efficacy of adverse outcomes in COVID-19 patients. In this study, COVID-19 patients with underlying diseases (**Table 1**), including diabetes, coronary heart disease, hypertension, and smoking history, were analyzed, with a higher proportion of diabetes in the severe group compared to the non-severe group. Patients with diabetes are not only more susceptible to the SARS-COV-2 but also tend to experience immune imbalance, excessive inflammatory responses, and multi-organ dysfunction after infection, leading to more complex conditions and significantly increased risk of critical illness. There is research showing that cytokines closely related to cytokine storms (IL-6, TNF-α) and chemokines such as CXCL10 and CCL2 are significantly elevated in COVID-19 patients with diabetes, indicating that these patients are more prone to excessive inflammatory responses post-infection (37).

This study primarily focused on the expression characteristics of several cytokines and chemokines in the serum of COVID-19 patients and their potential clinical significance. By analyzing the differences in the expression of these factors in the serum of COVID-19 patients with different clinical prognoses and classifications, we initially clarified their relationship with the severity of COVID-19. Meanwhile, we analyzed the predictive value of related cytokines/chemokines for the prognosis of COVID-19 patients and their value in assessing the condition. Additionally, by tracking the dynamic changes in cytokines/chemokines in some patients and exploring their correlation with lymphocyte subpopulations, we preliminarily elucidated their potential clinical significance in the progression of COVID-19.

There are limitations to this study. Firstly, our sample size was limited, and we did not observe the relationship between sCD25 and T cell counts. Moreover, the dynamic monitoring data of cytokines/chemokines, especially in surviving COVID-19 patients, were limited. Therefore, future studies should use larger sample sizes and collect more longitudinal data to further deepen our research. Secondly, we lacked a healthy control group, and the inclusion of healthy control data may enhance the completeness of our study results. Finally, the relevant mechanisms of cytokines/chemokines in the pathological process of COVID-19 were not addressed in this study and require further validation *in vitro* experiments. Studies have shown that vaccination can significantly improve outcomes for COVID-19 patients (38, 39). However, a limitation of this study was the failure to collect vaccination records of the patients. Therefore, future studies should consider collecting the important data to provide a more comprehensive analysis and further understand the role of this variable in COVID-19.

## Data Availability

The original contributions presented in the study are included in the article/supplementary material. Further inquiries can be directed to the corresponding author.
